# Risk Factors for Unplanned Emergency Department Visits in Patients with Nasopharyngeal Carcinoma During Radiotherapy

**DOI:** 10.3390/biomedicines12112616

**Published:** 2024-11-15

**Authors:** Wei-Shan Chen, Chien-Lin Lee, Wei-Chih Chen, Ching-Nung Wu, Tai-Jan Chiu, Yao-Hsu Yang, Hao-Wei Lu, Sheng-Dean Luo, Yu-Ming Wang

**Affiliations:** 1 Department of Otolaryngology, Kaohsiung Chang Gung Memorial Hospital, College of Medicine, Chang Gung University, Kaohsiung 833, Taiwan; b0002066@cgmh.org.tw (W.-S.C.); jarva@cgmh.org.tw (W.-C.C.); taytay@cgmh.org.tw (C.-N.W.); 2 Department of Hematology-Oncology, Kaohsiung Chang Gung Memorial Hospital, College of Medicine, Chang Gung University, Kaohsiung 833, Taiwan; b9902018@cgmh.org.tw (C.-L.L.); kuerten@cgmh.org.tw (T.-J.C.); 3 Department of Public Health, College of Medicine, National Cheng Kung University, Tainan 701, Taiwan; 4 Graduate Institute of Clinical Medical Sciences, College of Medicine, Chang Gung University, Taoyuan 333, Taiwan; 5 School of Traditional Chinese Medicine, College of Medicine, Chang Gung University, Taoyuan 333, Taiwan; gmailr95841012@adm.cgmh.org.tw; 6 Department of Traditional Chinese Medicine, Chang Gung Memorial Hospital, Chiayi 613, Taiwan; 7 Health Information and Epidemiology Laboratory, Chang Gung Memorial Hospital, Chiayi 613, Taiwan; 8 Department of Radiation Oncology, Jen-Ai Hospital, Taichung 412, Taiwan; jahn140@mail.jah.org.tw; 9 School of Medicine, College of Medicine, National Sun Yat-Sen University, Kaohsiung 804, Taiwan; 10 Department of Radiation Oncology & Proton and Radiation Therapy Center, Kaohsiung Chang Gung Memorial Hospital, College of Medicine, Chang Gung University, Kaohsiung 833, Taiwan

**Keywords:** nasopharyngeal carcinoma, radiotherapy, chemoradiation, emergency, outcomes

## Abstract

**Background/Objectives:** Nasopharyngeal carcinoma (NPC) is commonly treated with radiotherapy (RT) or concurrent chemoradiotherapy (CCRT). However, unplanned emergency department (ED) visits during treatment can disrupt therapy and impact patient outcomes. This study aims to identify the risk factors associated with unplanned ED visits in patients with NPC receiving RT or CCRT. **Methods:** We retrospectively analyzed 2111 patients with NPC treated between 2001 and 2019 at Chang Gung Memorial Hospital. Patients were categorized based on whether they experienced an unplanned ED visit during or up to three months post-treatment. Demographic and clinical variables were compared using the Chi-squared test, and survival outcomes were assessed using Kaplan-Meier analysis. **Results:** Among the cohort, 573 patients (27.2%) experienced at least 1 unplanned ED visit. Risk factors for unplanned ED visits included older age (*p* < 0.001), hypertension (*p* < 0.001), higher Charlson Comorbidity Index (*p* = 0.001), and advanced clinical stage (T stage, *p* = 0.0046; N stage, *p* = 0.0034; M stage, *p* = 0.0008). No significant difference in ED visit rates was observed between RT alone and CCRT groups. **Conclusions:** Unplanned ED visits were common during NPC treatment, with risk factors primarily related to patient age, comorbidities, and disease stage. Identifying high-risk patients may enable interventions to reduce ED visits, improve survival outcomes, and alleviate healthcare costs.

## 1. Introduction

Radiotherapy (RT) is the first-line treatment for patients with nasopharyngeal carcinoma (NPC) due to its radiosensitive behavior. For advanced NPCs, the addition of chemotherapy to RT, concurrent chemoradiotherapy (CCRT), is proven to be superior in disease survival to radiotherapy alone [1,2,3,4,5]. In addition, there is substantial evidence showing survival benefits and impact on tumor control by adding induction chemotherapy to concurrent systemic therapy or RT [6,7]. Further studies and meta-analyses have proved the benefit of CCRT with and without adjuvant chemotherapy; it significantly improves survival and reduces locoregional or distant failure in regionally advanced NPC [8]. Generally, current evidence supports applying induction or adjuvant chemotherapy followed by concurrent systemic therapy or RT, compared to systemic therapy/RT alone, for locoregional advanced NPC. Induction chemotherapy may have a better result for controlling disease-distant progression compared to adjuvant chemotherapy [9,10,11,12,13,14]. However, adverse events and treatment toxicities may be seen during RT or CCRT. Acute side effects include dermatitis, mucositis, nausea and vomiting, neutropenia, and impaired hepato-renal function. Patients who received CCRT were reported to have more acute severe treatment toxicities, such as acute mucositis and acute nausea or vomiting, than radiotherapy alone [15].

Unexpected emergency department (ED) visits during cancer treatment can often disrupt treatment courses and impair compliance. The most common diagnoses for ED visits in patients with cancer were pneumonia, non-specific chest pain, urinary tract infection, and septicemia. Among them, pneumonia was the most common reason for cancer-related ED visits in adult patients and had a high rate of inpatient admission [16,17]. In addition, ED visits due to complications of systemic therapy or radiotherapy, such as neutropenia, sepsis, and anemia, could significantly increase the financial burden of the medical system [18]. Patients with head and neck squamous cell carcinoma (HNSCC) often need multi-disciplinary treatment, including surgery, radiotherapy, and chemotherapy. Prior studies have revealed that patients with HNSCC who received CCRT or RT had a significantly higher likelihood of unplanned ED visits and hospitalization during the treatment period [19,20,21]. However, those studies often excluded or did not focus on patients with nasopharyngeal carcinoma.

It is crucial to identify high-risk patients to optimize patient care and improve clinical outcomes. This study aims to identify high-risk factors contributing to unexpected ED visits during RT or CCRT treatment in patients with NPC and survey its impact on disease outcomes.

## 2. Materials and Methods

From 2001 to 2019, we retrospectively enrolled patients above 20 years old with newly diagnosed NPC who had undergone primary curative radiotherapy or concurrent chemoradiotherapy from Chang Gung Research Database (CGRD). Only patients having complete radiotherapy treatment records with available start-to-end dates were included. The need for informed consent was waived due to the retrospective study nature. Exclusion criteria include the following: age < 20 years old, incomplete radiotherapy treatment or missing records, patients with tracheostomy or feeding tubes, incomplete body mass index (BMI), and clinical TNM staging data. Patients with tracheostomy or feeding tubes were excluded because these groups of patients often have comorbidities other than NPC alone. Treatment intensity would often be lower and different from the dose given to patients with newly diagnosed NPC, hence causing bias when analyzed. We also excluded patients previously diagnosed with HNSCC because a great proportion of these patients have received radiotherapy before. Patients lacking complete BMI data and clinical TNM staging records were excluded to decrease potential bias. Demographic data including gender, age, BMI score, underlying comorbidities, Charlson comorbidity index score [22], disease clinical TNM stage, and treatment types were documented and analyzed. Patients were grouped as with or without unplanned ER visits from RT initiation to 3 months post-completion. In the group of patients with unplanned ER visits, their visit times were recorded. Overall survival is defined as the time from the new diagnosis of NPC to death of any cause at the last follow-up. We used the Kaplan-Meier test for survival analysis and the Chi-squared test for identifying risk factors for unplanned ER visits. A *p*-value < 0.05 was considered statistically significant.

## 3. Results

### 3.1. Patient Characteristics

**Figure 1:**
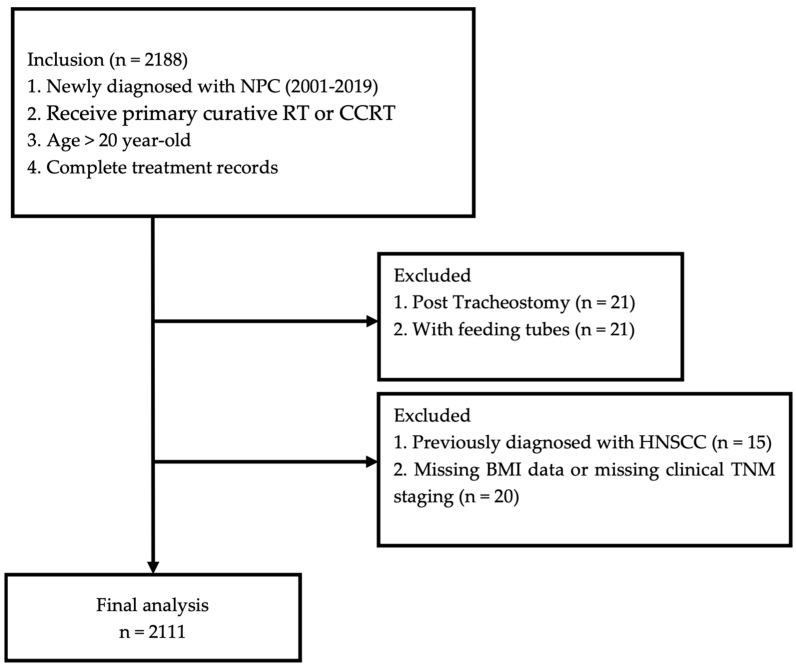
Flowchart of patient inclusion.

A total of 2188 patients with newly diagnosed NPC receiving primary RT or CCRT from 2001 to 2019 were included. After applying exclusion criteria, 2111 patients were enrolled in our analysis (Figure 1). Of these, 1604 (76%) were male and 507 (24%) were female. Sixty-six percent of the patients were under 55 years old. Ninety percent of the patients received concurrent systemic therapy for cancer treatment. A total of 1892 (90%) patients had comorbidities of hypertension, but 78% of them had a Charlson Comorbidity Index (CCI) score of zero. A total of 48.6% of patients had clinical T3 and T4 tumors. For clinical N stage, N1 was observed in 810 patients (38.4%), N2 in 575 patients (27.2%), and N4 in 444 patients (21%). The detailed patient characteristics along with disease staging are shown in Table 1.

### 3.2. ED Visit Time Point in Patients with NPC

**Figure 2:**
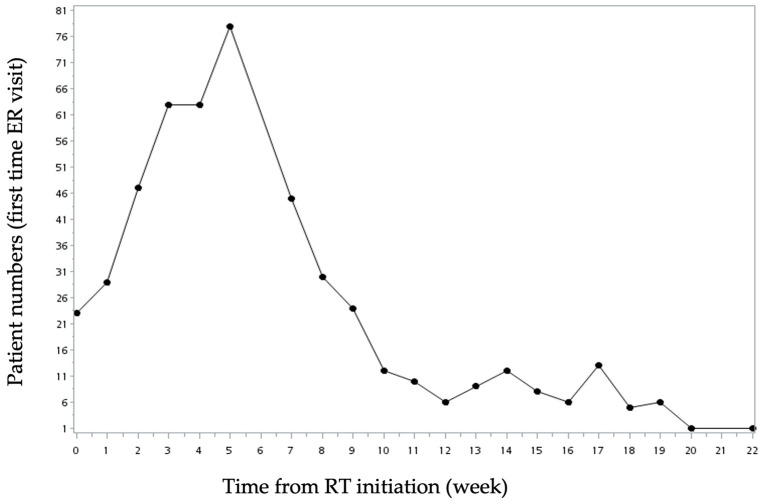
Patient numbers of first-time ED visits at different time points after RT initiation.

Among all patients, 573 (27.2%) experienced unexpected ED visits from the start of RT until 3 months post-treatment completion. Of these, 363 (63.35%) had single ED visits, 134 (23.39%) had two visits, and 76 (13.26%) had more than three ED visits (Table 2). We further analyzed the time point of the first unexpected ED visit in each patient. Twenty-three (4%) patients had their first ED visit in the first week of RT initiation. The number of patients gradually increased every week as RT went on and reached its peak at 5–6 weeks after RT initiation when 27.9% of patients had their first visits (Figure 2). The number of patients then decreased after RT completion.

### 3.3. Survival Analysis and Risk Factors for Unplanned ED Visits

**Figure 3:**
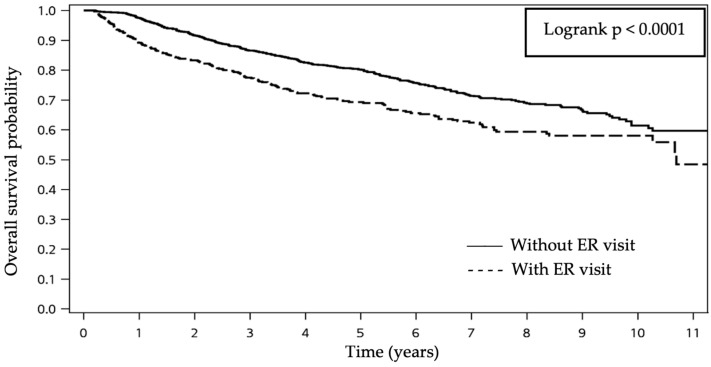
Kaplan-Meier survival curves of overall survival for patients with NPC, with versus without ED visits.

For survival analysis, we found that patients who experienced unplanned ED visits had significantly poorer overall survival, with a median survival time of 10.6 years (*p* < 0.001, Figure 3). Risk factors for unplanned ED visits were evaluated. We identified that patients with unplanned ED visits are more likely to have older age (*p* < 0.001), underlying comorbidities with hypertension (*p* < 0.001), a higher CCI-score (*p* = 0.001), advanced clinical T stage (*p* = 0.0046), advanced clinical N stage (*p* = 0.0034), and advanced clinical M stage (*p* = 0.0008). Meanwhile, there was no significant difference in the rate of unplanned ED visits between patients receiving RT plus concurrent systemic chemotherapy or RT alone (Table 3).

## 4. Discussion

Our study revealed that nearly one-third of patients diagnosed with nasopharyngeal carcinoma (NPC) and undergoing curative RT or CCRT experienced unplanned ED visits from the start of treatment to three months post-treatment. The majority of these ED visits occurred during the RT course, particularly peaking at 5–6 weeks after initiation. This timing suggests a critical period when patients are most vulnerable to treatment-related complications. Furthermore, those who had unplanned ED visits exhibited significantly poorer overall survival outcomes compared to their counterparts who did not require emergency care. The identified risk factors contributing to these unexpected medical needs included older age, the presence of underlying comorbidities, and advanced disease status, all of which highlight the importance of targeted monitoring and intervention strategies for at-risk patients during their treatment journey. Understanding these dynamics is crucial for optimizing patient care and improving clinical outcomes in this population.

Patients experiencing unplanned ED visits during cancer treatment often present with a variety of common complaints. These include pain, respiratory distress, nausea and vomiting, and fever. Such symptoms can be indicative of complications arising from both the cancer itself and the side effects of treatment modalities like RT and CCRT. The need for immediate medical attention during treatment is significant; a substantial proportion of these ED visits ultimately result in hospitalization [16,17], which can complicate the patient’s treatment course and overall care trajectory. For patients diagnosed with head and neck squamous cell carcinoma, numerous studies have highlighted an elevated risk of experiencing unplanned medical events during treatment. A large cohort study indicated that a considerable proportion—ranging from 28% to 55%—of patients with HNSCC would have ED visits or unexpected admissions during their cancer treatment. Among these patients, those receiving CCRT alone or those who underwent surgery followed by CCRT exhibited even higher risks of requiring emergency medical attention [19]. This trend underscores the challenges faced by individuals undergoing aggressive cancer therapies, where treatment-related toxicities can lead to acute medical needs.

In a specific cohort analyzed by Moore et al. [20], it was reported that 36% of patients with HNSCC experienced unplanned ED visits during their treatment period. The majority of these visits were attributed to toxicities associated with systemic therapy and radiotherapy, manifesting as symptoms like nausea, vomiting, pain, and mucositis. These side effects significantly impact a patient’s quality of life and may necessitate immediate medical intervention. Additionally, Tang et al. [23] described the characteristics of ED visits among patients with HNSCC in Taiwan, noting that 31.14% of their cohort visited the ED once, while 29.46% required further admission after their initial visit. However, it is important to note that many of these studies did not specifically include patients with nasopharyngeal carcinoma (NPC), which is a distinct subgroup within head and neck cancers.

To the best of our knowledge, our study represents the first comprehensive evaluation of unplanned ED visits and their impact on patients specifically diagnosed with NPC. Our findings indicated that 27.2% of patients with NPC experienced unexpected ED visits during their treatment, a statistic that aligns closely with similar reports concerning patients with HNSCC. Notably, we observed that up to 13.26% of these patients had multiple ED visits, with some experiencing more than three during their treatment course. Up to 13.26% of these patients even had ED visits more than three times. Moreover, our analysis revealed that the peak time for the first unplanned emergency room (ER) visit occurred between 5 and 6 weeks after the initiation of radiotherapy (RT), which is still within the treatment period. This timing is particularly concerning, as it suggests that patients are experiencing significant complications during a critical phase of their therapy. Such adverse events can lead to interruptions in treatment, which may ultimately impair patient outcomes and overall survival rates. The findings from our study highlight that a substantial proportion of patients with NPC face unexpected medical needs during their treatment journey. Identifying these patients early is crucial for optimizing their care and implementing timely interventions. By recognizing those at higher risk for unplanned ED visits, healthcare providers can develop targeted strategies to mitigate complications, enhance patient support, and ensure continuity of care throughout the treatment process. This proactive approach is essential for improving the overall management of NPC and achieving better health outcomes for affected individuals.

Risk factors associated with adverse events that lead to unplanned medical interventions in patients with HNSCC have been documented in various studies. Advanced disease stages, specifically stage III and IV, along with a Charlson Comorbidity Index (CCI) score of 1 or higher, have been consistently identified as independent predictors of non-cancer-related health events, unplanned hospitalizations, or emergency department (ED) visits among patients with HNSCC [19,24]. These factors reflect the complexity of managing patients with more advanced diseases, where both the tumor burden and comorbidities pose significant challenges to maintaining treatment continuity. Ling et al. [25] further noted that specific comorbidities, such as diabetes and pre-existing pulmonary diseases, alongside an increased radiation dose, significantly contributed to prolonged hospital stays in patients with HNSCC during or within 8 weeks following RT. These findings underscore the vulnerability of this patient population to treatment-related toxicities, particularly in those who present with a higher burden of pre-existing conditions or who receive aggressive therapeutic regimens. High-risk patients with HNSCC often experience a range of post-treatment complications. Commonly observed adverse events include aspiration, radiation-induced airway edema, dehydration, poor nutritional intake due to mucositis, severe nausea/vomiting, and febrile neutropenia [11,25]. These complications not only have the potential to interrupt scheduled treatments, which may compromise treatment efficacy, but they also contribute to increased healthcare utilization through unplanned ED visits or hospital admissions. The impact of these interruptions is multifaceted, affecting not only clinical outcomes, such as disease control and overall survival, but also significantly increasing the financial burden on healthcare systems.

In the context of NPC, treatment-related adverse effects similarly include dermatitis, mucositis, nausea, vomiting, and neutropenia, which may necessitate additional medical attention during or after RT and CCRT. Our study supports these findings, as significant risk factors for unplanned ED visits in patients with NPC included older age, higher CCI scores, and more advanced TNM staging. These patients are particularly vulnerable during the critical phases of their treatment, when the cumulative effects of radiotherapy and chemotherapy may exacerbate underlying conditions or lead to severe treatment-related toxicities. Notably, previous studies have highlighted that multimodal treatment regimens, such as CCRT, tend to increase the likelihood of adverse events and unexpected medical needs compared to RT alone [19,24]. However, our study cohort demonstrated no significant difference in the rate of unplanned ED visits between patients with NPC treated with CCRT and those treated with RT alone. This lack of difference could be due to several factors, including the inherent differences in baseline characteristics such as age, comorbidity burden, and tumor biology between patients with NPC and HNSCC. Moreover, patients with specific cancer subsites, such as laryngeal or hypopharyngeal cancer, are known to have a higher risk of treatment-related complications, particularly respiratory and gastrointestinal symptoms, compared to NPC or cancers in other head and neck regions [19]. This distinction may partly explain why patients with NPC, despite undergoing aggressive treatments, do not exhibit a markedly higher incidence of unplanned ED visits compared to their HNSCC counterparts. Understanding these complexities is crucial for developing personalized care strategies aimed at minimizing unplanned medical interventions and optimizing outcomes for high-risk patients with NPC or other head and neck cancers. Proactive risk assessment and timely management of adverse events could reduce the frequency of treatment interruptions and improve long-term clinical outcomes in this vulnerable population.

Koch et al. [26] reported that approximately one-fourth of patients with cancer who visited the ED due to cancer-related complications exhibited significantly worse overall survival than those whose visits were unrelated to cancer. This highlights the severe impact of cancer-specific complications on survival outcomes. In a study of patients with Taiwanese colorectal cancer, those with ED visits had a 5-year overall survival rate of 0.56; however, there was no significant difference in survival when comparing patients with and without ED visits [27]. This indicates that the impact of ED visits on survival may vary by cancer type, with some cancers showing a more pronounced effect. In patients with HNSCC, a particularly poor survival outcome was noted, with about 18% of patients dying within 30 days of their ED visit [23]. This high mortality rate underscores the importance of managing complications such as infection, mucositis, and respiratory distress, which often lead to emergency medical needs in these patients. Our study similarly revealed that patients with NPC who experienced unplanned ED visits during their treatment course had significantly worse overall survival compared to those who did not. This aligns with the broader understanding that unplanned medical interventions are indicative of severe treatment-related toxicities or pre-existing health conditions that complicate the patient’s recovery. The need for early identification and management of high-risk patients is essential to improving clinical outcomes. By adopting preventive strategies, such as closer monitoring or proactive symptom management, healthcare providers can reduce the likelihood of ED visits and potentially improve survival rates for patients with NPC.

Moreover, the economic burden associated with unplanned ED visits is substantial. These visits, particularly those related to complications from CCRT or radiotherapy RT, often lead to extended hospital stays and higher healthcare costs [25]. By identifying patients at higher risk for such complications, targeted interventions can be implemented to reduce the need for emergency care, thereby alleviating the financial strain on healthcare systems and improving the overall efficiency of cancer care.

Our study has several limitations. First, it was a retrospective study and included only single-center data. Second, due to the relatively high accessibility of medical resources in Taiwan’s health system, the reasons for visiting the ED could be various and might not accurately report the extent of true medical needs. Future studies should focus on the cause of these ED visit events in patients with NPC to more precisely evaluate their impact on disease outcomes. Lastly, early identification of these high-risk patients and further care strategies to prevent these events are mandatory. Possible methods include close monitoring by case managers on the phone or in person, increasing times for outpatient clinic visits, or enhancing education about early signs and symptoms of treatment adverse effects for patients and families. A previous cohort study reported implementing a weekly symptom management clinic for high-risk patients with HNSCC who were undergoing RT [28]. Their result showed a reduction in ED visits, unplanned hospital admissions, and medical costs. Setting up appropriate care and preventive strategies for this group of high-risk patients will be our future point.

## 5. Conclusions

Our study is by far the first to highlight the characteristics and burden of unplanned ED visits among patients with NPC undergoing RT or CCRT. Nearly a third of patients experienced unplanned ED visits, with the peak incidence occurring between 5 and 6 weeks of the RT treatment period. Risk factors identified include older age, underlying comorbidities, and advanced disease stages. Future efforts should focus on developing prevention methods for high-risk patients in order to improve disease outcomes and reduce the economic burden on the healthcare system.

## Figures and Tables

**Table 1:** Patient demographics and characteristics.

Variables		Patient (Total n = 2111)
**Gender**		
	Male	1604 (76%)
	Female	507 (24%)
**Age group**		
	≦55	1398 (66%)
	56–65	476 (23%)
	65–75	190 (9%)
	>75	47 (2%)
**BMI group**		
	<18.5	65 (3%)
	18.5–24	840 (40%)
	≧24	1206 (57%)
**Concurrent Systemic Therapy**		
	Without	209 (10%)
	With	1902 (90%)
**Hypertension**		
	Without	1892 (90%)
	With	219 (10%)
**CCI-score group**		
	0	1641 (78%)
	1	236 (11%)
	2	99 (5%)
	≧3	135 (6%)
**Clinical T stage**		
	I–II	1085 (51.4%)
	III	489 (23.2%)
	IV	537 (25.4%)
**Clinical N stage**		
	0	282 (13.4%)
	I	810 (38.4%)
	II	575 (27.2%)
	III	444 (21%)
**Clinical M stage**		
	0	2039 (97%)
	1	72 (3%)
**Death**		507 (24%)

CCI: Charlson Comorbidity Index.

**Table 2:** Distribution of ED visit times and patient number.

ED Visit Times	Patient Number (Total n = 573)
1	363 (63.35%)
2	134 (23.39%)
≧3	76 (13.26%)

**Table 3:** Risk factors for unplanned ED visits.

Variable		ED Visit		*p*-Value
		Without (n = 1538)	With (n = 573)	
**Gender**				0.8742
	Male	1170 (72.9%)	434 (27.1%)	
	Female	368 (72.6%)	139 (27.4%)	
**Age group**				**<0.0001**
	≦55	1058 (75.7%)	340 (24.3%)	
	56–65	340 (71.4%)	136 (28.6%)	
	65–75	116 (61.1%)	74 (38.9%)	
	>75	24 (51.1%)	23 (48.9%)	
**BMI group**				0.1617
	<18.5	49 (75.4%)	16 (24.6%)	
	18.5–24	593 (70.6%)	247 (29.4%)	
	≧24	896 (74.3%)	310 (25.7%)	
**Concurrent Systemic Therapy**				0.0546
	Without	164 (78.5%)	45 (21.5%)	
	With	1374 (72.2%)	528 (27.8%)	
**Hypertension**				**<0.0001**
	Without	1405 (74.3%)	487 (25.7%)	
	With	133 (60.7%)	86 (39.3%)	
**CCI-score group**				**0.0001**
	0	1234 (75.2%)	407 (24.8%)	
	1	155 (65.7%)	81 (34.3%)	
	2	67 (67.7%)	32 (32.3%)	
	≧3	82 (60.7%)	53 (39.3%)	
**Clinical T stage**				**0.0046**
	1/2	824 (75.9%)	261 (24.1%)	
	3	341 (69.7%)	148 (30.3%)	
	4	373 (69.5%)	164 (30.5%)	
**Clinical N stage**				**0.0034**
	0	224 (79.4%)	58 (20.6%)	
	1	606 (74.8%)	204 (25.2%)	
	2	401 (69.7%)	174 (30.3%)	
	3	307 (69.1%)	137 (30.9%)	
**Clinical M stage**				**0.0008**
	0	1498 (73.5%)	541 (26.5%)	
	1	40 (55.6%)	32 (44.4%)	
**Death**		327 (64.5%)	180 (35.5%)	<0.0001

CCI: Charlson Comorbidity Index.

## Data Availability

The data presented in this study are available on request from the corresponding authors as the data are not publicly available due to privacy or ethical restrictions.
